# Cytocompatible Anti-microbial Dressings of S*yzygium cumini* Cellulose Nanocrystals Decorated with Silver Nanoparticles Accelerate Acute and Diabetic Wound Healing

**DOI:** 10.1038/s41598-017-08897-9

**Published:** 2017-09-05

**Authors:** Rubbel Singla, Sourabh Soni, Vikram Patial, Pankaj Markand Kulurkar, Avnesh Kumari, Mahesh S., Yogendra S. Padwad, Sudesh Kumar Yadav

**Affiliations:** 1 Nanobiology Lab, Biotechnology Division, CSIR-Institute of Himalayan Bioresource Technology, Palampur (H.P.), 176061 India; 2 Pharmacology and Toxicology Lab, Food and Nutraceuticals Division, CSIR-Institute of Himalayan Bioresource Technology, Palampur (H.P.), 176061 India; 3 Academy of Scientific and Innovative Research (AcSIR), CSIR-IHBT, Palmapur, India; 4 Center of Innovative and Applied Bioprocessing (CIAB), Knowledge City, Sector-81, Mohali, 140306 India

## Abstract

The ever increasing incidences of non-healing skin wounds have paved way for many efforts on the convoluted process of wound healing. Unfortunately, the lack of relevance and success of modern wound dressings in healing of acute and diabetic wounds still remains a matter of huge concern. Here, an *in situ* three step approach was embraced for the development of nanocomposite (NCs) dressings by impregnating silver nanoparticles (AgNPs) onto a matrix of cellulose nanocrystals (CNCs) isolated from *Syzygium cumini* leaves using an environmental friendly approach. Topical application of NCs (ointments and strips) on acute and diabetic wounds of mice documented enhanced tissue repair (~99% wound closure) *via* decrease in inflammation; increase in angiogenesis, collagen deposition, and rate of neo-epithelialization that ultimately led to formation of aesthetically sound skin in lesser time than controls. Due to the synergistic action of CNCs (having high water uptake capacity) and AgNPs (anti-microbial agents), NCs tend to increase the expression of essential growth factors (FGF, PDGF and VEGF) and collagen while decreasing the pro-inflammatory factors (IL-6 and TNF-α) at the same time, thus accelerating healing. The results suggested the potential of these developed anti-microbial, cytocompatible and nanoporous NCs having optimized AgNPs concentration as ideal dressings for effective wound management.

## Introduction

Acute dermal wounds heal quickly in healthy individuals but turn into deep sores in diabetics, leading to severe infections in underlying tissues^1^. Acute wounds follow an orderly healing process and close in a short span of time (~2 weeks), while diabetic wounds do not heal in an orderly manner and have a prolonged inflammatory phase, taking up to 3–4 weeks to recover^2^. The mechanisms underlying wound healing defects in diabetics are not fully understood, but may include deregulation of the biochemical milieu, altered growth factors, abnormal cytokine production, and high inflammation^3^. On a global scale, there is a huge demand of dermal wound dressings for diabetes related dermal infections^4^. Thereby, designing an ideal wound dressing is vital for promoting faster healing.

Basically, an ideal dressing must provide moist wound milieu, help to remove wound exudates and promote tissue regeneration besides being biocompatible and antimicrobial in nature^5, 6^. Advanced wound management involves use of gauze, hydrocolloids, hydrogels, foams, films, ionic silver impregnated dressings, and composite polymer dressings *viz*. cellulose, cellulose acetate, chitosan, polyvinyl alcohol etc.^7, 8^. Majority of these materials suffer from one or the other limitations such as non-biocompatibility, high cost, synthetic and infection prone nature, poor water absorption and difficulty in removal causing trauma to the healed skin^9^. Past reports have described the use of bacterial cellulose as wound dressings because of its high water absorption potential which aids in easy entrapment of exuding wound fluids^10, 11^. The major drawbacks behind the use of bacterial cellulose are its tedious isolation procedure, difficulty in culture maintenance for several days (~10 days), and affordability^12^. Also, nano-silver have been widely used as topical dressings due to their anti-microbial and anti-inflammatory properties^13, 14^, but the concentration dependent toxicity has always remained a matter of huge concern^15^. In-spite of all the endeavors, development of a perfect dressing still remains a challenging task owing to the profuse qualities required.

Towards this end, we report efforts to develop inexpensive, easily fabricated, absorbent, and biopolymer based nanocomposites (NCs) as dressings, fulfilling most of the desirable attributes for both acute as well as diabetic wound repair. In our recent publication, we reported the acute wound healing potential of NCs containing bamboo cellulose nanocrystals (CNCs) and AgNPs prepared from 1 mM solution of AgNO_3_^16^. CNCs isolated from different plant sources possess varied physico-chemical properties which could affect their healing potential, thereby, we have made an attempt to explore CNCs from an another plant source *Syzygium cumini* for its healing efficacy in acute and diabetic conditions. Novelty of this study lies in the use of an *in situ* green approach for the synthesis and simultaneous impregnation of AgNPs (formed by biological reduction of three different concentrations of AgNO_3_ solution) onto CNCs matrix to develop NCs (ointments and strips). Further, optimum concentration of AgNPs for anti-microbial and *in vivo* acute and diabetic healing potential was determined in mice through histolopathological, immunohistochemical and biochemical estimations. We hypothesize that nanoporous plant CNCs would aid in keeping the wound moist by controlling wound exudates due to their high water retaining capacity. This would further facilitate faster wound closure *via* regulation of certain growth factors and cytokines along with synergistic action of antibacterial and anti-inflammatory AgNPs.

## Results and Discussion

### Synthesis of Cellulose Nanocrystals (CNCs) and Nanocomposites (NCs)

Extraction of *S. cumini* CNCs (SC-CNCs) from *S. cumini* leaves using chemical treatments resulted in the isolation of pure cellulosic fibers by removal of lignin, pectin, and hemicelluloses^17^. Further an *in situ* approach was followed for NCs development where color change of reaction mixtures from transparent to brown implied the reduction of AgNO_3_ to AgNPs. Here, Ag ions from AgNO_3_ solution (1, 5, and 10 mM) were initially adsorbed onto SC-CNCs matrix *via* electrostatic or van der Waals forces and then Ag ions were reduced into AgNPs by the polyphenols and other biochemical constituents present in biological reducing agent i.e. *S. cumini* leaf extract (SC-LE). Due to quite inexpensive, environmental and human friendly nature, the use of biological reducing agent is favorable over other chemical reducing agents to develop NCs wound dressings. The images of NCs strip and ointment are shown in Supplementary Fig. S1.

### Microscopic Examinations Confirmed the Morphology of Materials

SEM showed the presence of smooth, porous, and ribbon shaped chemically pre-treated fibers (SC-CPFs) of average diameter in order of 10–25 μm, smaller than several hundred micrometers of rough bundles of untreated fibers (see Supplementary Fig. S2). This was mainly due to the effect of step-wise chemical pre-treatments that has induced disintegration of fiber bundles into individual fibers by solubilizing the non-cellulosic components^18^.

**Figure 1:**
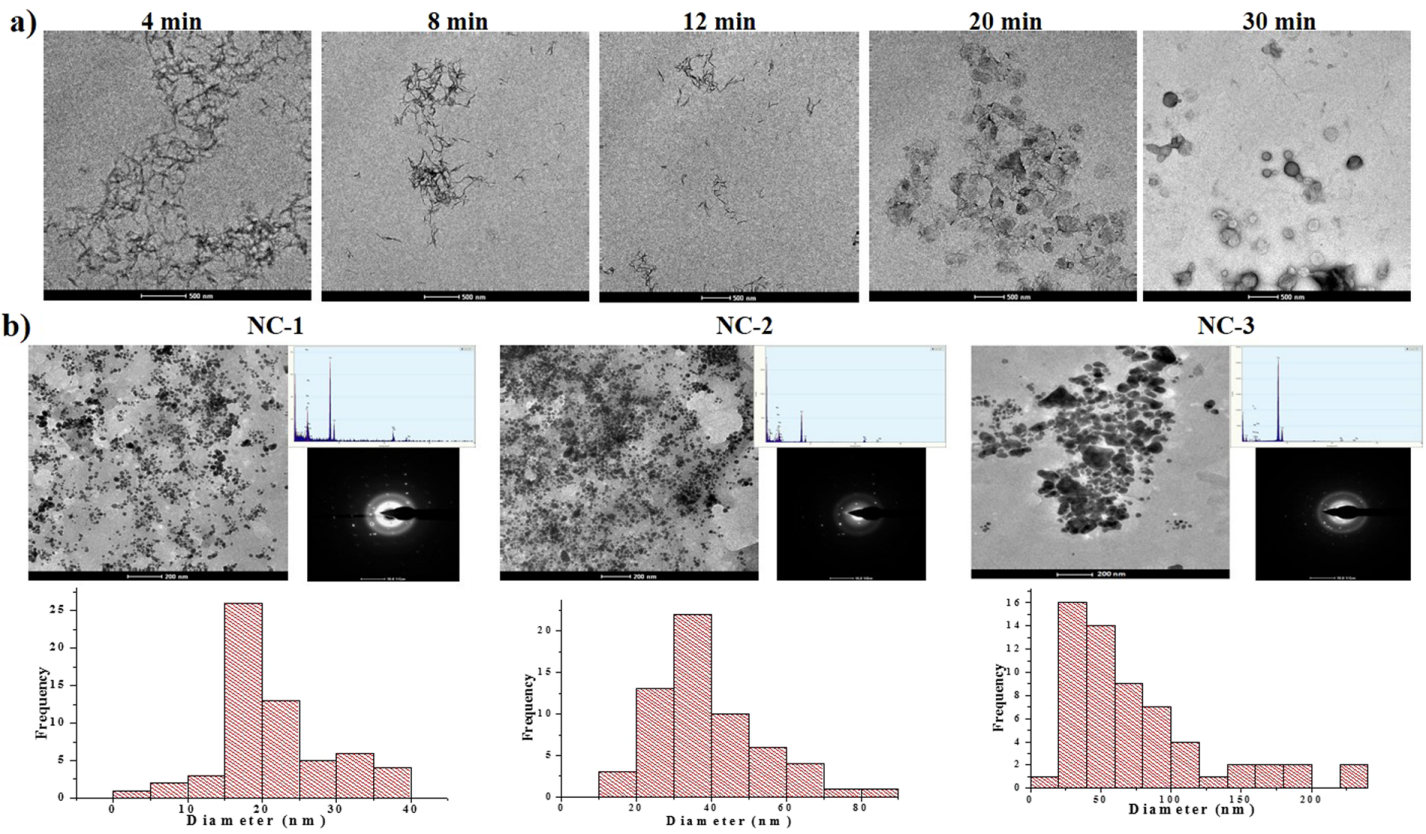
Transmission electron micrographs showing (**a**) morphology of SC-CNCs at variable sonication time points and (**b**) morphology, elemental composition, diffraction pattern, and size distribution histograms of NC-1, NC-2, and NC-3.

TEM micrographs exhibited the morphology of SC-CNCs obtained after sonication time periods of 4, 8, 12, 20 and 20 min. Average diameter of SC-CNCs was almost similar (16 ± 2 nm) in each case, whereas the length of rod shaped uniform SC-CNCs obtained after 4, 8, and 12 min of sonication was 225 ± 40, 210 ± 10, and 180 ± 20 nm, respectively. Acid hydrolysis caused shortening of length of SC-CPFs by breaking the bonds of amorphous regions present between crystalline regions to form SC-CNCs^18^. Spherical shaped cellulose nanoparticles started to form after 20 min sonication, while well-defined spheres of diameter 120 ± 20 nm were seen after 30 min (Fig. 1a). Spherical cellulose nanoparticles from waste cotton (diameter <100 nm) have already been reported after enzymatic hydrolysis and 30 min sonication^19^. Sonication for variable time periods resulted in defibrillation to individualize SC-CNCs, along with a distinctive change in morphology and length^17^. The variations in shape, surface area and nano-sizes of CNCs could affect the wound healing process^20^. SC-CNCs obtained after 12 min of sonication were selected further for NCs development as they possess definite shape (rods without any aggregations) and smallest size having largest surface area. Rod shaped CNCs with large surface area would be able to provide sufficient space (matrix) for impregnation of AgNPs during NCs development.

TEM micrographs showed the impregnation of spherical AgNPs (fillers) with mean diameter 21 ± 7 nm, and 40 ± 14 nm onto SC-CNCs matrix to form NC-1 and NC-2, respectively whereas agglomerated AgNPs of variable shapes with 70 ± 30 nm size were formed in NC-3 (Fig. 1b). Histograms representing size distribution of AgNPs indicated higher frequency of small sized AgNPs in NC-1, followed by NC-2, and broad size ranged AgNPs in NC-3. This might be due to the fact that with increase in concentration of precursor salt, greater number of AgNPs are formed in the same volume of solution which finds a limited space to bind on SC-CNCs matrix and ultimately creates higher coalescence^21^. During the formation of NCs, SC-CNCs form a 3-D network upon which AgNPs bind through strong ion-dipole interactions of Ag^+^ with carboxyl and hydroxyl groups of SC-CNCs leading to stabilization of AgNPs^22^. Biochemical constituents of SC-LE and surface hydroxyl groups of SC-CNCs might be responsible for reducing AgNO_3_ salt to AgNPs, controlling their size distribution and stabilization onto SC-CNCs matrix^23, 24^. TEM-EDX showed the peaks of elemental Ag indicating the existence of AgNPs on SC-CNCs, whereas patterns of selected area electron diffraction (SAED) demonstrated the concentric diffraction rings as bright spots corresponding to different crystal planes, revealing the polycrystalline nature of NCs (Fig. 1b). Enlarged TEM images of NCs and TEM-EDX are presented in Supplementary Fig. S3. TEM image of bare AgNPs (without CNCs) used as control is given in Supplementary Fig. S4.

### Zeta Potential Studies Documented Surface Charges and Stability of Aqueous Dispersions of SC-CNCs

Aqueous suspension of SC-CNCs displayed a mean value of −35 ± 5 mV (see Supplementary Fig. S5). Negative charge implied the existence of surface functional moieties such as carboxyl, hydroxyl, and sulfates on SC-CNCs as a result of acid hydrolysis that led to fairly stable dispersion of CNCs in water^25^. Average zeta potentials of NC-1, NC-2, and NC-3 were −25 ± 2, −20 ± 2, and −12 ± 4 mV, respectively. Decrease in negative zeta potential of NCs as compared to SC-CNCs was possibly due to the interaction of AgNPs with the surface moieties of SC-CNCs. The successive decrease in negative zeta potential values from NC-1 to NC-3 indicates the aggregation of AgNPs, which might be due to the increase in number of AgNPs^26^.

### Spectroscopic Analysis of NCs

UV-Vis spectra of NCs showed a characteristic peak ranging 420–480 nm, attributed to surface plasmon resonance of AgNPs confirming their impregnation onto SC-CNCs matrix (see Supplementary Fig. S6). Peak broadening and red shift with relatively lower intensity was observed with increase in concentration of AgNO_3_ from NC-1 < NC-2 < NC-3. Increase in concentration of AgNO_3_ also caused agglomeration and settling of AgNPs impregnated onto SC-CNCs that could be responsible for lowering of peak intensity^21^. The concentration of Ag determined by atomic absorption spectroscopy (AAS) was found to be ~0.067, 0.19, and 0.4 wt% in NC-1, NC-2, and NC-3 ointments, whereas NC-1, NC-2, and NC-3 strips contained ~0.9, 2.54, 5.4 wt% of Ag, respectively.

FT-IR spectra confirmed that non-cellulosic components were removed after chemical treatments during the isolation of SC-CNCs (see Supplementary Fig. S7). It has also indicated an interaction of −OH groups of SC-CNCs with AgNPs present in NCs. Details of all the changes noticed in peak intensity at particular wavenumber of each sample is mentioned in supplementary information.

XRD spectra of untreated leaf, SC-CPFs and SC-CNCs showed three well-defined peaks around 2θ = 16°, 22.5° and 34.5° highlighting the crystalline nature of cellulose with a sharp peak at 22.5° corresponding to 002 lattice plane (see Supplementary Fig. S8). Intensity scattered by the amorphous part was measured as lowest intensity at 16–18°. Untreated leaf, SC-CPFs and SC-CNCs possessed %CI values of 52, 67 and 75%, respectively. Increase in %CI of SC-CPFs than that of untreated leaf was due to the removal of lignin and hemicelluloses during chemical pre-treatments^27^. Dissolution of amorphous region by acid hydrolysis further increased %CI of SC-CNCs. The crystallinity arises from the organized arrangements of crystalline portions of plant nanocellulose, illustrating the structure of cellulose-I^27^. Not much difference was noticed in %CI of plant cellulose and bacterial cellulose (~83%)^22^. Small but significant peaks at 38°, 44°, 64° and 77° were attributed to face centered cubic crystalline structure of AgNPs in NCs^28^. Peak intensity at 22.5° decreased in NCs with an increase in concentration of AgNPs which could be due to increase in interactions of SC-CNCs and AgNPs with increase in concentration.

### Mechanical Strength of NCs

The tensile strength of NC-1, NC-2, and NC-3 strips was calculated to be 0.013 ± 0.003, 0.038 ± 0.007, and 0.047 ± 0.005 MPa, respectively. It was noticed that with increase in wt% of fillers (AgNPs) in NCs, the tensile strength increases. Similar ranged (0.02–0.05 MPa) tensile strength has been reported for nano ZnO coated chitin hydrogel composite bandages and it was suggested to be more than sufficient for a wound dressing material^29^. The results have affirmed the use of developed NCs as optimal wound dressing biomaterials.

### Water Uptake Capacity of NCs as Wound Dressing Materials

A wound dressing biomaterial should favor the absorption of wound exudates and maintain a certain level of moisture around the wound bed to facilitate healing by preventing dehydration of the tissue^30^. In this context, measurement of water uptake (%) showed that SC-CNCs absorbed a maximum of 300 ± 12% water, whereas NC-1, NC-2 and NC-3 absorbed 268 ± 10, 206 ± 8 and 118 ± 5% of water in 2 h followed by an equilibrium phase (see Supplementary Fig. S9). According to past report, water uptake (%) of bamboo CNCs was in the range of 141–171%^16^, regenerated cotton cellulose has shown water uptake of 129% in 0.5 h^31^. Water molecules easily penetrate in nano-structural pores of SC-CNCs due to its hydrophilic behavior. Lesser water uptake capacity of NCs in relation to SC-CNCs might be attributed to the filling of micro-voids of nanocellulose by small-sized AgNPs^32^. Water uptake capacity of NCs enables them to entrap exudates and to keep the wound site moist which is desirable for their effectiveness as wound dressing.

### Anti-Microbial Activity of NCs and its Mechanistic Action for Inhibiting Microbial Growth around Wound

Experiment to analyze the effect of different NCs (difference in terms of concentration and sizes of AgNPs) on anti-microbial activity is necessary to prove the applicability of NCs as wound dressings. Bacteria exposed to NCs (ointments and strips) showed zone of inhibition (ZOI), in contrast to bacteria exposed to control SC-CNCs only (see Supplementary Fig. S10). The measurements of ZOI against each bacterium are presented (see Supplementary Table S1). Differences in growth inhibition of each bacterium were observed, indicating that the anti-microbial action depends on the structural and chemical composition of bacterial cell membrane^33^. NC-1 showed maximum ZOI representing remarkable inhibitory effect on bacterial growth followed by NC-2 and NC-3, respectively. Microbicidal activity could be due to the diffusion of AgNPs from NCs to the culture medium. Large number of small sized spherical AgNPs present in NC-1 (21 ± 7 nm) showed greater anti-microbial action in comparison to relatively larger sized spherical AgNPs of NC-2 (40 ± 14 nm) and agglomerated variable shaped particles of NC-3 (70 ± 30 nm). The results illustrated that the anti-microbial efficacy of NCs was dependent more on the size rather than concentration of AgNPs that could be due to the greater tendency of small sized spherical AgNPs with larger surface area to enter the bacterial cell wall and cause cell death^34^. Recently, AgNPs prepared from *Piper longum* fruit extract having an average diameter of 46 nm at 20 µg/mL showed ZOI of 14 and 18 mm against *S. aureus* and *P. aeruginosa* ^35^. Similarly, AgNPs (20 nm) prepared from *Justicia adhatoda* L. resulted in ZOI of 7–9 mm against *P. aeruginosa* ^36^.

**Figure 2:**
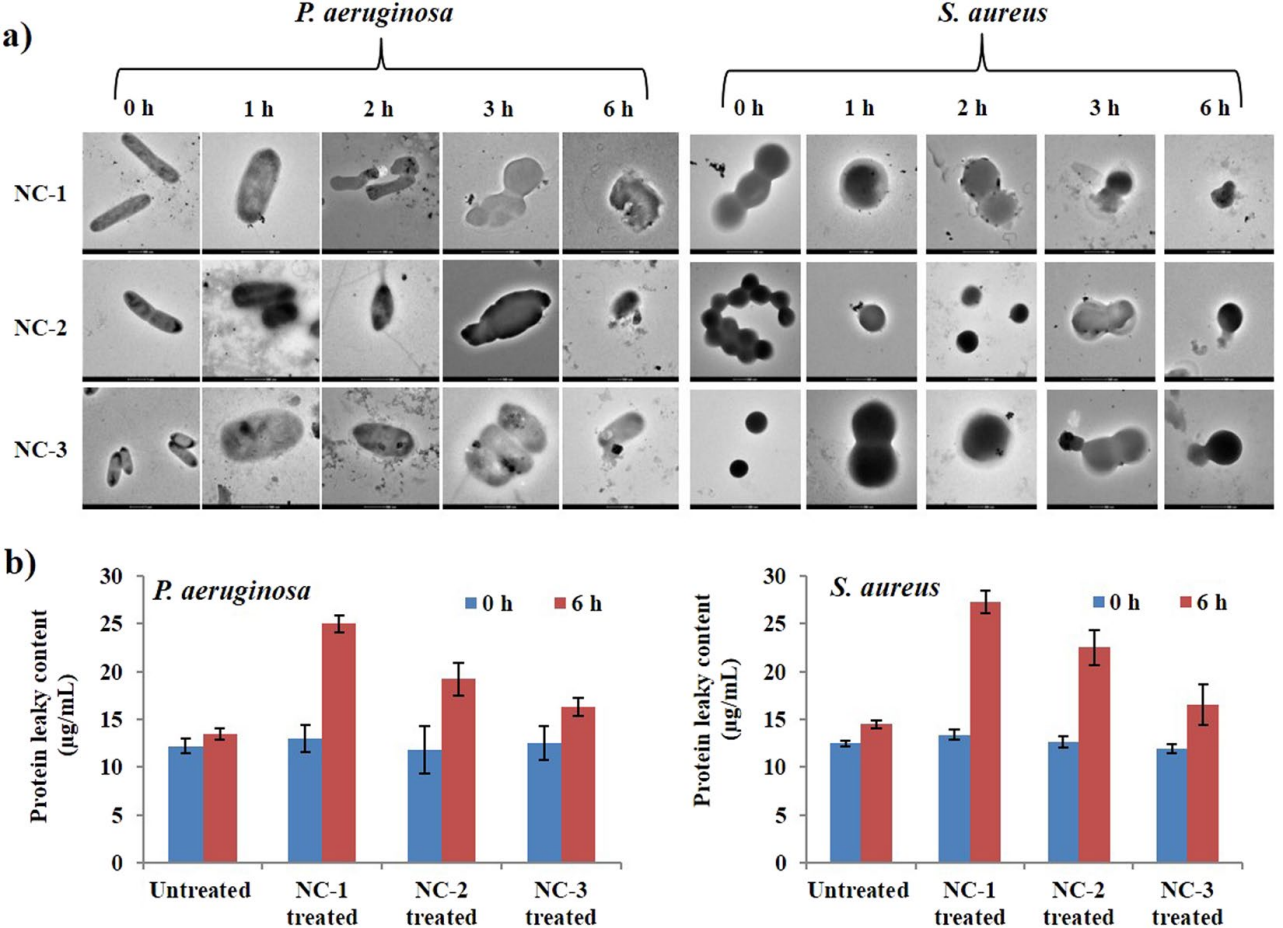
(**a**) Transmission electron microscopic images showing the change in morphology of *P. aeruginosa* and *S. aureus* after incubation with NC-1, NC-2 and NC-3 for different time intervals (0, 1, 2, 3 and 6 h) and (**b**) graphs showing protein leaky content of untreated and treated bacteria, *P. aeruginosa* and *S. aureus* at 0 h and 6 h.

Morphological changes in bacteria were observed at 0, 1, 2, 3 and 6 h of incubation with all the NCs (Fig. 2a). Initially at 0 h, *S. aureus* was round whereas *P. aeruginosa* was rod shaped. After 2 h of incubation, AgNPs from NC-1 showed adherence to bacterial membrane followed by penetration inside it. Membrane deformity and oozing out of intracellular components was visualized following 3 h of incubation, leading to the formation of cellular debris and cell death at 6 h of incubation with NC-1. The same sequence of events was also visualized in case of NC-2 and NC-3 but time lag was noticed. At 6 h of incubation, bacterial cells have shown membrane damage after exposure to NC-3. This might be due to the reason that after a decrease in number of small sized AgNPs with increase in concentration of Ag from NC-1 < NC-2 < NC-3, extent of particle penetration inside bacterial membrane decreases. The toxicity to bacteria also decreases in order of NC-1 > NC-2 > NC-3 as small sized NPs cause greater toxicity due to their significantly large surface area that come in contact with bacteria. Similarly, a previous report has shown the effect of AgNPs sizes (5–100 nm) on bactericidal action^34^.

Furthermore, protein leaky content measured from NCs treated and respective untreated bacterial cells was nearly similar at 0 h (Fig. 2b). After 6 h of incubation, a noteworthy increase in the protein leakage was observed from cells treated with NCs. Protein leakage was more in NC-1 followed by NC-2 and NC-3 treated bacteria. This could be due to the reason that small sized AgNPs possess larger surface area, come in contact with bacteria, as well as release more Ag ions, which promote interactions with the cells^37^. These positively charged Ag ions might interact with macromolecules present in biological systems through protein thiol groups (-SH), and inactivate proteins leading to cell death^38^. Although the exact mechanism of anti-bacterial action is still known, but from our results, we can state that small sized AgNPs comes in contact/penetrates bacterial cell surface, further releasing more Ag ions that lead to distortion of cell membrane and release of intracellular contents, resulting in cell death^39^. From the anti-bacterial mechanistic studies, it can be clearly stated that irrespective of concentration of precursor salt used for AgNPs preparation, their size effect was highly pronounced to influence the anti-microbial potential of NCs. Overall, the experimental outcomes accounted for the immense potential of these anti-microbial NCs as wound dressings.

### *In Vitro* Cytocompatibility Analysis Documented the Safety of NCs against Primary Mice Keratinocytes

AgNPs are known to be toxic for eukaryotes in a dose dependent manner. Thereby, cytocompatibility (%) measurement of prepared NCs was done by sulforhodamine B (SRB) assay in order to assess their safety against keratinocytes for use as wound dressings. The results showed that even after 48 h of treatment with 25 mg/mL of each NCs sample, keratinocytes showed ~60–70% cellular viability (in a concentration dependent manner), whereas positive control (vinblastine) showed <30% cellular viability after 48 h of treatment (see Supplementary Fig. S11). High percentage of cell viability in mice keratinocytes after exposure to NCs provides novel opportunities for the safe application of these biocompatible NCs as topical wound dressings. Thereby, 25 mg of each NCs ointment having highest viability (~75% after 24 h exposure) was considered an optimal dose and thereby applied daily on the dorsal surface of wounded mice to assess their healing potential.

### Visual Examination of Wounded Mice Model Documented the Enhanced Healing Potential of NCs

**Figure 3:**
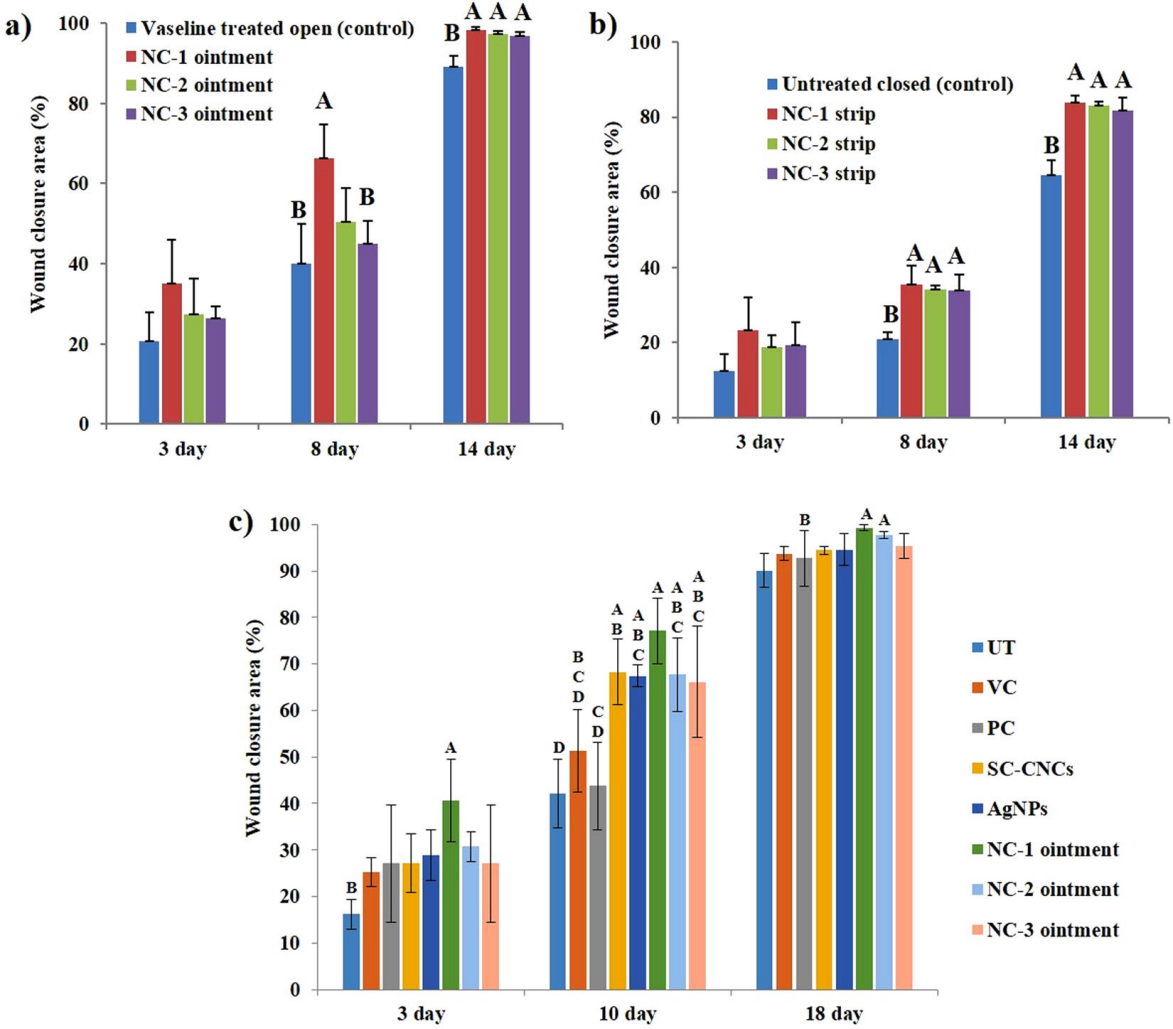
Histograms showing wound closure area (%) of NCs treated and control groups (**a**,**b**) at day 3, 8 and 14 post wounding in acute wounds of mice, and (**c**) at day 3, 10 and 18 in wounds of diabetic mice. The measurements are presented as mean ± standard deviation, n = 4 mice/group/timeline. Significant differences between NCs treated and control groups are indicated by different alphabets (p < 0.05).

Wound closure is evaluated as reduction in the wound area with respect to time and is efficiently assessed *in vivo* *via* quantitative measurement of wound area at specified time intervals^40^. Experimental data set indicating original morphology of wounds and how the wound size reduced over time in NCs treated swiss albino mice as compared to their respective controls at day 3, 8 and 14 (acute) and at day 3, 10 and 18 post-wounding (diabetic) has been showcased (see Supplementary Figs S12, S13 and S15). Significant differences (p ≤ 0.05) between mean wound closure area (%) in all the groups after statistical analysis by one way ANOVA have been exhibited (Fig. 3 and Table S2). On day 3, no evidence of infection with slight wound closure was discerned in treatment groups whereas marked inflammation was ascertained in control groups. At day 8 (acute) and 10 (diabetes) post-injury, the wounds of NCs treated mice had already lost their scab and appeared completely epithelialized, whereas wounds in control groups showed only partial re-epithelialization with prominent scab. At day 14 (acute) and 18 (diabetes) post wound, signs of redness and wound fluidity were still evident in case of control groups. Statistical analysis revealed that NC-1 treated mice showed maximum wound closure in both acute as well as diabetic mice model.

Our results have shown that NCs ointments have shown faster rate of wound closure than NCs strips which could be possibly due to higher gaseous and fluid exchange in ointment treated wounds. The superior healing effect of NC-1 as compared to NC-2 and NC-3 could be due to the smaller size and larger surface area of AgNPs irrespective of concentration in the former^41^. Amazingly, NC-1 (ointment and strip) containing significantly lesser Ag concentration as compared to AgNPs functionalized bacterial cellulose (2.62 wt%)^22^ acted as an effective bactericidal agent as well as possessed high water uptake capacity due to the presence of SC-CNCs, leading to better and rapid healing in about two weeks^42^. As NCs ointments have shown better and faster healing as compared to NCs strips in acute wounds, thus we carried out the diabetic study to test the efficacy of NCs ointments only.

### Histopathological Analysis Documented Enhanced Granular Tissue Formation, Angiogenesis, Collagen Deposition and Neo-Epithelialization Following NCs Treatment

**Figure 4:**
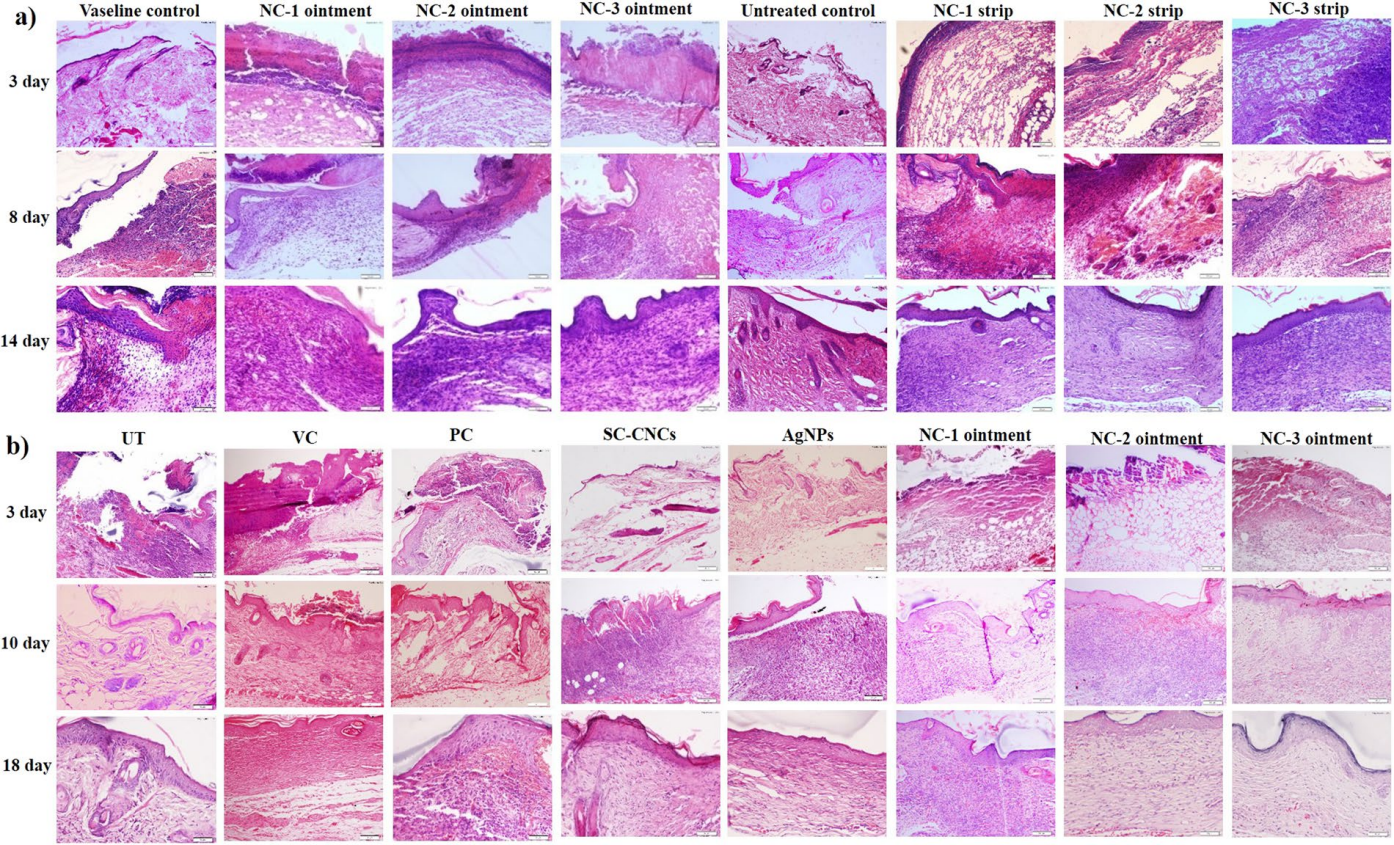
Bright field micrographs of hematoxylin and eosin (H&E) stained wounded skin tissue sections determining the changes in healing events in NCs treated and control mice groups (**a**) at day 3, 8 and 14 post wound in acute wound study and (**b**) at day 3, 10 and 18 post wound in diabetic wound study.

Histopathological data revealed that at the outset of healing process (day 3), wound margins were easily demarcated by an abrupt interruption in continuity between epithelium and dermis. Initiation of neo-epithelialization was observed beneath the scab tissue in treatment groups. H&E stained sections at day 3 in all the groups depicted a hefty influx of neutrophilic granulocytes at wound site but their number was lesser in NCs treated groups as compared to respective controls (Fig. 4 and Fig. S15). Downsized inflammation in all the treatment groups might be due to the large number of carboxylate groups of SC-CNCs which hold a higher concentration of Ag. Anti-inflammatory effect of AgNPs promotes wound healing by inhibition of both macrophage infiltration as well as release of inflammatory cytokines^43^. Differences among the groups were semi-quantitatively analyzed on the basis of inflammation, angiogenesis, fibroplasia and re-epithelialization in H&E stained skin tissue sections of acute and diabetic mice and are presented in Table 1.

**Table 1:** Semi quantitative analysis based on histopathological findings representing wound healing sequence of events in all mice groups after topical application of treatment materials, where symbols represented as: +++ = Abundant, ++ = Moderate, + = Scanty, − = Nil.

Mice treatment groups		Mice treatment groups			Extent of angiogenesis			Amount of fibrous tissue formation			Re-epithelialization rate		
		Day 3	Day 8	Day 14	Day 3	Day 8	Day 14	Day 3	Day 8	Day 14	Day 3	Day 8	Day 14
**Acute wound healing**	**Vaseline open control**	+++	++	++	−	++	++	−	+	+	−	+	++
	**NC-1 ointment**	++	−	−	−	+++	+++	−	++	+++	−	++	+++
	**NC-2 ointment**	++	+	−	−	++	++	−	++	++	−	++	+++
	**NC-3 ointment**	+++	++	+	−	+	++	−	+	++	−	+	++
	**Untreated closed control**	+++	++	++	−	++	++	−	+	++	−	+	++
	**NC-1 strip**	++	++	+	−	++	+++	−	++	+++	−	++	+++
	**NC-2 strip**	++	++	+	−	+	++	−	+	+	−	++	+++
	**NC-3 strip**	++	++	+	−	++	++	−	+	++	−	+	++
**Diabetic wound healing**		**Day 3**		**Day 10**	**Day 10**		**Day 18**	**Day 10**		**Day 18**	**Day 10**		**Day 18**
	**UT**	**+++**		**++**	**+**		**++**	**+**		**+**	**+**		**++**
	**VC**	**+++**		**++**	**+**		**++**	**+**		**++**	**+**		**++**
	**PC**	**++**		**++**	**+**		**++**	**+**		**++**	**+**		**+++**
	**SC-CNCs**	**++**		**++**	**+**		**++**	**+**		**++**	**+**		**++**
	**AgNPs**	**+++**		**++**	**++**		**++**	**+**		**++**	**+**		**++**
	**NC-1 ointment**	**++**		**+**	**+++**		**+++**	**++**		**+++**	**++**		**+++**
	**NC-2 ointment**	**++**		**+**	**++**		**+++**	**++**		**+++**	**++**		**+++**
	**NC-3 ointment**	**++**		**++**	**++**		**++**	**++**		**+++**	**+**		**+++**

At day 8 (acute) and 10 (diabetes), accelerated healing was observed in NCs treated mice relative to the control groups which still harbored prominent signs of inflammation (Fig. 4a,b). The neo-epithelial layer from marginal wound edges was thick, connected to the underneath matrix and showed initiation of granulation tissue organization in NCs treated groups. NCs-treated mice documented fibroblast migration, and well-organized compact collagen bundles in dermis, but control groups showed the presence of pale and randomly oriented collagen fibrils as illustrated by M&T staining (see Supplementary Fig. S14a and b). Nanoporous structure of SC-CNCs is beneficial for cell migration and proliferation^6^. NCs were able to decrease inflammation, bolster fibroblast migration and collagen deposition in dermis more efficiently than control groups marking the transition to the proliferative phase of healing. Similar findings were observed in acute wound healing study by our group using bamboo NCs^16^. The probable reason behind this finding could be the protease inactivating property of AgNPs resulting in decreased inflammation, thereby reducing time for granulation tissue formation^44^.

At day 14 (acute) and 18 (diabetes), efficient wound repair was observed in NCs treated groups as demarcated by reconstitution of dermal-epidermal junction. The neo-epidermis by this time point covered a much more mature bedding of dermal tissue (Fig. 4). Blood vessels and well organized compact collagen fibers parallel to epidermis were seen in NCs treated groups relative to less developed and randomly organized collagen fibrils in control groups (see Supplementary Figs S14 and S15). AgNPs present in NCs seem to quicken repair process by differentiation of fibroblasts into myofibroblasts^43^. NCs promoted rapid re-epithelialization along with development of some hair follicles that signified the movement of tissue repair process towards completion. This could be accredited to the property of CNCs to provide moist environment in the wound milieu thereby hastening healing^45^. Furthermore, the extent of collagen synthesis was considerably higher in NC-1 ointment treated groups as compared to other ointment and all the strip treated groups.

### Immunohistochemistry (IHC) in Skin Tissues of Diabetic Mice

**Figure 5:**
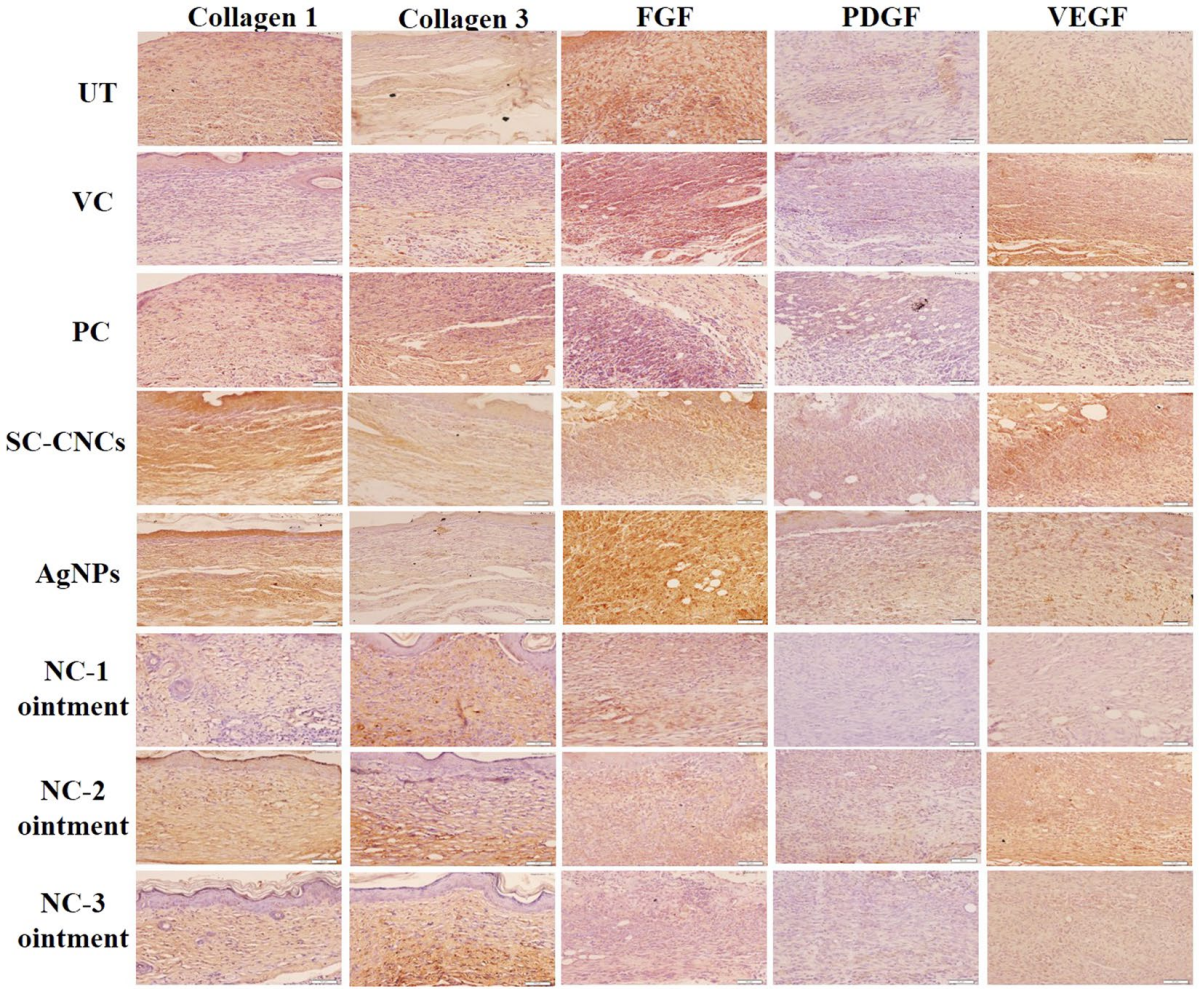
Representative immunohistochemical stained images of wound tissue sections of diabetic mice on day 10 (FGF, PDGF, VEGF) and day 18 (collagen 1 and 3) post wounding. Brown color represents positive immunostaining against specific proteins observed in epidermal and dermal layers having an uneven distribution.

Growth factors such as platelet-derived growth factor (PDGF), basic fibroblast growth factor (b-FGF) and vascular endothelial growth factor (VEGF) act as potential mediators in healing by accelerating chemotaxis, migration, stimulation and proliferation of fibroblasts, endothelial cells and other factors that regulate the local wound environment^46^. The level of expression of collagen 1, collagen 3, PDGF, FGF and VEGF were determined in wound tissue sections through IHC (Fig. 5 and see Supplementary Fig. S14a–e).

The balance between collagen synthesis and degradation is tenuous in diabetic wound repair where its synthesis is markedly decreased, resulting in healing complications^47^. As evident from IHC images, expression of collagen is significantly (p < 0.05) higher in NCs treated diabetic mice as compared to control groups (see Supplementary Fig. S16). Previous reports have evidenced the use of polysaccharide based dressings for increasing collagen density in wounds^48^. Levels of PDGF have been shown to be lower in diabetic wounds. NCs are believed to enhance wound healing through increase in PDGF expression. Our study results have shown that NC-1 treated diabetic mice groups have significantly higher expression of PDGF in comparison to all other groups (see Supplementary Fig. S16). Previous reports have documented that absence of FGF lead to delayed healing in diabetic wounds^49^. Higher levels of FGF were observed in NCs treated groups suggesting the role of NCs in accelerating wound repair process via FGF up-regulation. In diabetics, angiogenesis has been shown to be impaired affecting vessel formation and healing possibly due to involvement of VEGF^50^. Statistical analysis of our results has shown a significant increase in VEGF levels upon NC-1 treatment as compared to untreated control which has resulted in intensified angiogenesis and accelerated healing (see Supplementary Fig. S16). The stimulation and accumulation of these factors might be occurring as a result of elevated proteolytic activity in the presence of moist wound environment due to high water uptake capacity of SC-CNCs^51^.

### Biochemical Estimations of Serum/Skin Homogenate of Mice

**Figure 6:**
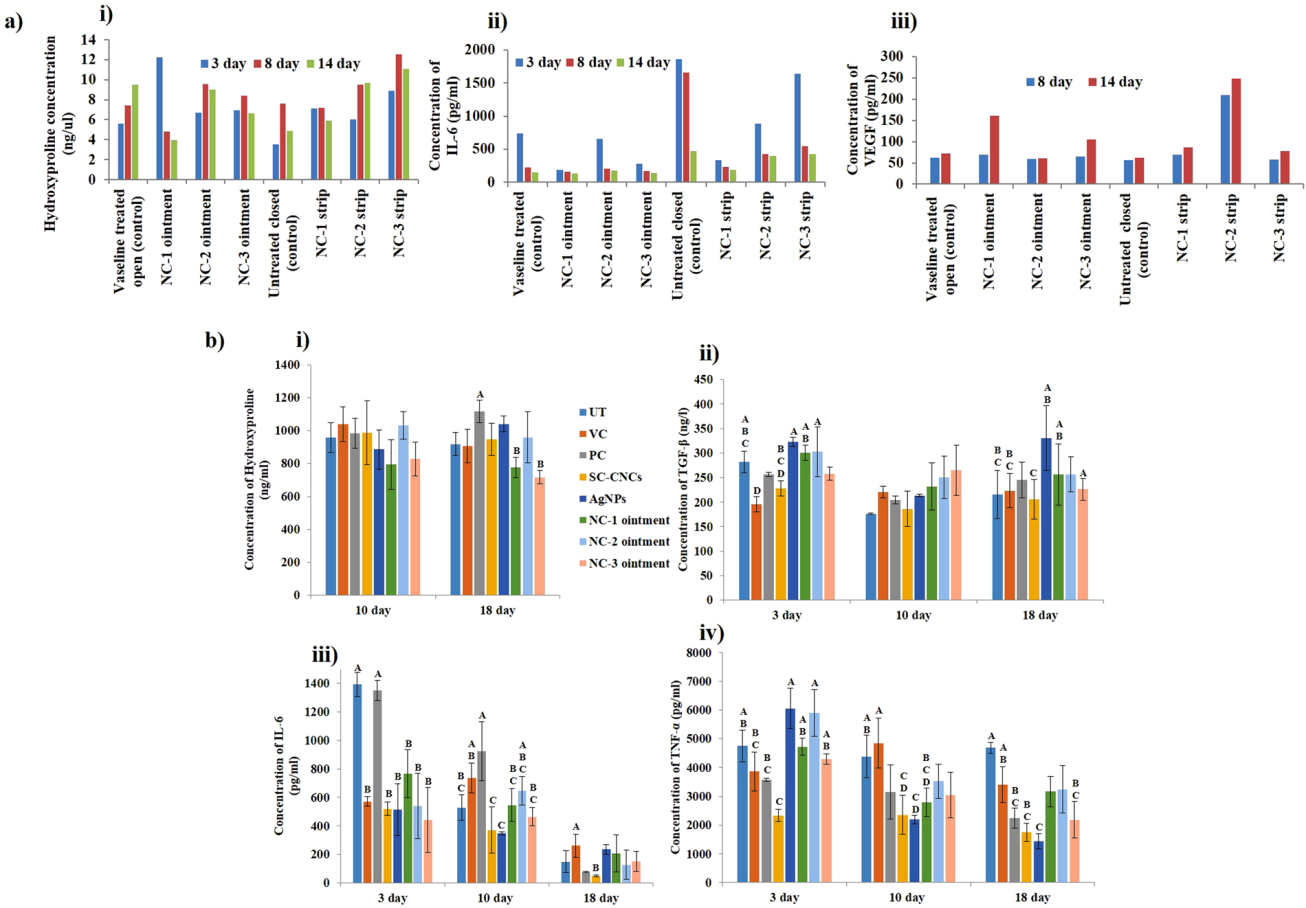
Biochemical estimations in (**a**) serum of acute wound mice model quantifying the levels of (i) hydroxyproline, (ii) IL-6 and (iii) VEGF; (**b**) skin homogenates of diabetic mice model to assess (i) hydroxyproline, (ii) TGF-β, (iii) IL-6 and (iv) TNF-α levels at specific time points post wound.

The collagen content estimation in wounded tissue was done by the assessment of hydroxyproline content, a major constituent of collagen. Level of collagen was found to be enhanced at day 8 than day 3 in treated acute wounds as compared to control groups (Fig. 6 and Table S2). Subsequently, a decline in collagen content was observed at day 14 in all the treated groups. By day 14 post-wounding, treated groups have entered a phase of dermal remodeling where equilibrium was achieved between the rate of collagen formation and degradation^43^. Similar results were obtained in diabetic wounds where hydroxyproline levels in NCs treated groups decreased at day 18 in comparison to day 10.

IL-6, a pro-inflammatory cytokine, plays crucial role in modulating immune responses and is a requisite for timely wound repair^52^. High levels of IL-6 were observed in all the groups at day 3. Concentration of IL-6 decreased in treated groups by day 8 post-wounding which further depreciated by day 14 as compared to control groups (Fig. 6 and Table S2). The resulting reduction in inflammation was accredited to anti-inflammatory nature of AgNPs^41^. In normal wound healing the highest levels of pro-inflammatory cytokine, TNF-α are seen from 12 to 24 h after wounding. At low levels, TNF-α contributes to the stimulation of fibroblasts and keratinocytes, the expression of growth factors and up-regulation of antimicrobial defense but shows detrimental effects on healing at higher levels^53^. TNF-α levels are elevated in diabetes in part through increased oxidative stress that promotes inflammation. Our results have validated the fact that NCs treatment has led to decrease in TNF-α levels resulting in faster and efficient healing in diabetic wounds (Fig. 6). This might be due to the inhibitory effect of AgNPs on the release of IL-6 and TNF-α^54^.

VEGF is a major player in promoting angiogenesis, vasculogenesis and closure in excision wounds^55^. At day 8 post-treatment, control groups displayed considerably lesser serum levels of VEGF than that of NCs treated acute wounds (Fig. 6 and Table S2). At day 14, VEGF levels were found to be increased in treated groups. Results suggested that NCs treatment has enhanced VEGF production, brought about intensified angiogenesis and accelerated healing. TGF-β is an important regulator of the ECM as it stimulates fibroplasia and collagen deposition. It also has a role in inhibiting ECM degrading proteases and up-regulating the synthesis of protease inhibitors^50^. Overproduction of TGF-β is induced by hyperglycemia in diabetes which in turn stimulates production of collagen, fibronectin and proteoglycans. Our findings have shown that NCs treatment has no significant effect on TGF-β levels in diabetic mice (Fig. 6).

## Conclusions

In the present study, NCs (ointment and strip) embodying plant nanoporous SC-CNCs functionalized with AgNPs were prepared using three different concentrations of AgNO_3_ by following an environmental friendly *in situ* approach. NCs having optimized concentration of AgNPs exhibited excellent cytocompatibility, anti-microbial, high water uptake and anti-inflammatory properties. NC-1 ointment showcased best results in terms of early neo-vascularization as well as enhanced collagen deposition, and faster re-epithelialization that ultimately led to better and rapid healing through synergistic effects of AgNPs (lower concentration and smaller size) and SC-CNCs. Thus, these NCs possess immense potential to be used as topical dressing for treatment of acute as well as diabetic dermal wounds in future.

## Materials and Methods

### Isolation of cellulose nanocrystals (CNCs)

CNCs from the leaves of *Syzygium cumini* (SC) were isolated by a combination of chemical treatments (bleaching (NaClO_2_), alkali (KOH) and acid hydrolysis (H_2_SO_4_)) followed by mechanical (ultrasonication) treatment adopting a procedure detailed in supporting information. The fibers obtained after bleaching and alkali treatment were deemed as chemically pre-treated fibers (CPFs). SC-CPFs after acid hydrolysis and ultra-sonication were abbreviated as cellulose nanocrystals (CNCs). The aqueous suspension of SC-CNCs was characterized and then lyophilized.

### Preparation of nanocomposites (NCs) in strip and ointment forms

The leaf extract (LE) of SC used for synthesis of NCs was prepared using our previously published method^20^. NCs development comprised of one pot approach harboring three steps. In the first step, 100 mg of already isolated SC-CNCs (1 wt%) were suspended individually in 10 mL of each AgNO_3_ solution (1, 5, and 10 mM) and sonicated for 2 min. Secondly, LE (10% v/v) was added and the reaction mixture was stirred for 6 h at room temperature. Finally, the mixture was centrifuged at 10,000 rpm for 10 min. The pellet obtained was washed repeatedly with distilled water to remove unbound salts. After centrifugation, the pellets obtained consisted of SC-CNCs impregnated with AgNPs termed as NCs hydrogels. NCs (strips) were prepared by dissolving the NCs hydrogels in water, followed by casting in a mold of desirable shape and size, and drying in hot air oven (35 °C). To prepare NCs (ointments) as topical dressing, NCs hydrogels were further mixed with Vaseline^®^ (inert base) in 1:1 ratio. NCs prepared using SC-CNCs and AgNPs from AgNO_3_ (1, 5 and 10 mM) were designated as NC-1, NC-2, NC-3, respectively. NCs hydrogels were lyophilized to obtain a powder form for further characterization studies. A similar method was followed for the synthesis of bare AgNPs solution (without the incorporation of CNCs). These AgNPs alone were used as a control for animal studies.

### Characterization of SC-CNCs and NCs

The morphology of samples was examined by scanning electron microscopy (SEM Hitachi S-3400 N, Japan) and transmission electron microscopy (TEM Tecnai, Twin 200 KV, FEI, Netherlands). Energy dispersive X-ray spectroscopy (TEM-EDX) and selected area electron diffraction (SAED) was also performed. DLS zeta potential (Zetasizer Nano ZS, Malvern Instruments Ltd.) was carried out for surface charge estimations. UV-Vis absorption studies were undertaken using Nanodrop (UV-Vis spectrophotometer; ND-2000) at 300–800 nm. The amount of Ag present in NCs was determined by flame atomic absorption spectrometry (AAS, Analytik Jena, Vario-6). Fourier transform infrared spectroscopy (FTIR, Thermo Nicolet 6700) in the range of 400–4,000 cm^-1^ was performed. X-ray powder diffractometer (XRD-Rigaku, Japan) was used to calculate the percentage crystallinity index (%CI). The tensile strength testing of NC strips of dimensions (30 mm × 10 mm) was accomplished by texture analyzer (TA.XT Stable Micro System, UK).

### Calculation of water uptake capacity

Water holding capacity of SC-CNCs and NCs was measured in terms of water uptake (%). Samples were dried to a constant weight, cut into strips of identical size, weighed and then immersed in distilled water. At each specified time interval, strips were taken out and extra amount of water present at surface was blotted and weighed again. The same process was repeated until no further change in weight was observed. Water uptake (%) was calculated according to the formula given in eq. (1) where *W*_*o*_ is initial weight and *W*_*t*_ is the weight at time *t*.

1 $$Uptake\,of\,water\,( \% )=(\frac{{W}_{t}-{W}_{o}}{{W}_{o}})\times 100$$

### Evaluation of microbicidal activity of NCs and its mechanism

To study the effect of different concentrations and sizes of AgNPs present in NCs (ointments and strips) on anti-microbial activity, well diffusion and disc diffusion assays were performed, respectively. Pure strains of wound infecting gram negative bacteria *Pseudomonas aeruginosa* (MTCC No. 741), *Citrobacter freundii* (MTCC No. 8128), *Enterobacter cloacae* (MTCC No. 9125), and *Escherichia coli* strain BL-21, as well as few gram positive bacteria *Staphylococcus aureus* (MTCC No. 3160), *Staphylococcus epidermidis* (MTCC No. 435)*, Bacillus subtilis* (MTCC No. 121) and a fungus *Candida parapsilosis* (MTCC No. 4448) were selected. The anti-bacterial mechanism of action of NCs was studied by observing a change in bacterial morphology and protein leaky content of a gram positive (*S. aureus*) and a gram negative (*P. aeruginosa*) bacteria after incubation with NCs^38^. The detailed methodology followed is mentioned in supporting information.

### *In vitro* cytocompatibility evaluation of NCs

Keratinocytes were isolated from 2 day old swiss albino mice following the procedure reported by Lichti *et al*.^56^. Experimental procedures were carried out as mentioned in the protocol approved by the Institutional Animal Ethics Committee (IAEC; Approval no. IHBT3-MAR2015) of CSIR-IHBT, Palampur based on guidelines of the federal regulatory agency, Committee for the Purpose of Control and Supervision of Experiments on Animals (CPCSEA; Registration no. 1381/GO/ReBiBt/S/2010/CPCSEA, Dt: 14/03/2016), Government of India. Sulforhodamine B (SRB) assay was conducted against viable keratinocytes grown in RPMI media to measure cytocompatibility of NCs following the procedure detailed in supplementary information.

### *In vivo* acute and diabetic wound healing studies

Swiss albino mice, 6–8 weeks old (27–35 g) were procured from our in-house experimental animal facility. Experimental procedures were carried out as mentioned in the protocol approved by the IAEC (Approval no. IHBTP-11/2014 and IHBT6-MAR2015) of CSIR-IHBT, Palampur based on guidelines of the federal regulatory agency, Committee for the Purpose of Control and Supervision of Experiments on Animals (CPCSEA; Registration no. 1381/GO/ReBiBt/S/2010/CPCSEA, Dt: 14/03/2016), Government of India. The mice were kept on *ad libitum* diet and categorized according to the topically applied wound dressing materials into 9 groups (6 mice/group) for acute wound healing as described in Table 2. For diabetic wound healing study, streptozotocin (STZ) was administered to mice for diabetes induction according to the procedure detailed in supporting information. Diabetic mice were then randomly divided into 8 groups (9 mice/group) as described in Table 2.

**Table 2:** Categorization of mice groups according to given biomaterial treatment.

Sr. No.	Mice groups according to material applied (Acute wound healing)	Mice groups according to material applied (Diabetic wound healing)
1.	Vaseline treated open control	Untreated diabetic control (UT)
2.	NC-1 ointment	Vaseline (vehicle) control (VC)
3.	NC-2 ointment	Betadine (positive) control (PC)
4.	NC-3 ointment	SC-CNCs control
5.	Untreated closed control	AgNPs control
6.	NC-1 strip	NC-1 ointment
7.	NC-2 strip	NC-2 ointment
8.	NC-3 strip	NC-3 ointment
9.	AgNPs alone (control)	—

For wounding, the dorsal skin of animals was shaved and sterilized with 5% povidone/iodine solution. The mice were anaesthetized using ketamine/xylazine mixture (90 mg/kg and 5 mg/kg body weight, respectively) administered intra-peritoneally and an excision wound of 0.5 cm^2^ (8 mm diameter) on dorsal surface was created using a circular biopsy punch. For treatment in acute wound healing, NCs (ointments) were applied daily on each wound (50 mg/wound/day), while strips were changed every 3^rd^ day over the study period of 14 days. In case of diabetic wounds, the efficacy of NCs ointments (but not strips) was investigated for a study time period of 18 days.

### Visual and histopathological analysis of wounded mice

To validate healing events, wound site was photographed, and wound diameter was measured with the help of a standard scale/vernier caliper for the quantification of wound closure area (%) on day 3, 8 and 14 (acute) and day 3, 10 and 18 (diabetic) post-wounding. At these time points, mice from each group (1 and 3 mice in acute and diabetic study, respectively) were euthanized; full thickness wound tissues along with marginal normal skin were excised, followed by immediate fixation in 10% neutral buffered formalin. Fixed tissues were further paraffin-embedded and cut into 5-μm-thick sections. The sections were stained with hematoxylin and eosin (H&E) and investigated microscopically^57^. To further enumerate the extent of healing, a semi-quantitative grading approach was adopted. Furthermore, the sections were also stained with Masson’s trichome (M&T) stain for gauging deposition of collagen fibers.

Immunohistochemistry (IHC) was also performed to study the expression of important factors playing a role in wound healing paradigm. Specific antibodies against platelet-derived growth factor (PDGF), basic fibroblast growth factor (b-FGF), vascular endothelial growth factor (VEGF), collagen I and collagen III were used in IHC to investigate their expression in wounded skin tissue sections of diabetic mice. Quantitative expression of each factor was determined from IHC images using ImageJ software. The details of methodology followed for IHC is given in supporting information.

### Biochemical analysis

In acute wound study, before euthanizing the mice, blood was collected *via* retro-orbital plexus for serum separation. The serum amassed from each mice group at day 3, 8 and 14 post-wound was estimated for hydroxyproline (indirect biochemical marker of collagen), IL-6 (interleukin-6, a pro-inflammatory cytokine) and VEGF (a proliferative phase marker) levels. Collagen was quantified using colorimetric based hydroxyproline assay kit (MAK008-Sigma-Aldrich, USA) whereas IL-6 and VEGF levels were determined using ELISA kits (RAB0308 and RAB0509, Sigma-Aldrich, USA) following manufacturer’s protocol.

In case of diabetic study, wound tissue collected at each timeline (3, 10 and 18 days post wound) was homogenized in 0.1 M PBS and then centrifuged at 10000 rpm for 15 min at 4 °C. The obtained skin homogenates were biochemically estimated for hydroxyproline, IL-6, TGF-β (transforming growth factor) and TNF-α (tumor necrosis factor) levels using ELISA (following manufacturer’s protocol). The absorbance was taken at specified wavelength to calculate the amount of these factors at a particular time interval to study the effect of NCs treatment on the wound healing events.

### Statistical analysis

The synthesis and characterization of all the nanomaterials and experiments related to anti-microbial activity and cytotoxicity were performed at least three times to confirm data reproducibility. The results are presented as mean ± standard deviation calculated from a large number of particles. For animal studies, comparison and data analysis between control and NCs treated groups for quantification of wound closure area (%) was done using JMP software (SAS Institute). The significance of difference was assessed with one way ANOVA; statistical significance level was set at p < 0.05. In acute study, the number of mice per group euthanized at each timeline was not large enough (as permitted by IAEC) to be used for applying statistics in serum biochemical assays.

## Electronic supplementary material

Supplementary Information
