# Supplementary material for: Cytocompatible Anti-microbial Dressings of Syzygium cumini Cellulose Nanocrystals Decorated with Silver Nanoparticles Accelerate Acute and Diabetic Wound Healing
Source: Sci Rep. 2017 Sep 5;7:10457. doi: 10.1038/s41598-017-08897-9 (PMC5585312; doi:10.1038/s41598-017-08897-9)
Supplement: Supplementary file 1 — Supplementary Information [file 41598_2017_8897_MOESM1_ESM.doc]

**Supplementary information**

**Cytocompatible Anti-microbial Dressings of S*yzygium cumini* Cellulose Nanocrystals Decorated with Silver Nanoparticles Accelerate Acute and Diabetic Wound Healing**

**Rubbel Singla^1,3,¥^, Sourabh Soni^2,3,¥^, Vikram Patial^2,3^, Pankaj Markand Kulurkar ^2^, Avnesh Kumari^1,3^, Mahesh S.^2^,Yogendra S. Padwad^2,3^,*& Sudesh Kumar Yadav^1,3,4^,***

^¥^ Equal contribution

^1^Nanobiology Lab, Biotechnology Division, CSIR-Institute of Himalayan Bioresource Technology, Palampur (H.P.) – 176061, India.

^2^Pharmacology and Toxicology Lab, Food and Nutraceuticals Division, CSIR-Institute of Himalayan Bioresource Technology, Palampur (H.P.) – 176061, India.

^3^Academy of Scientific and Innovative Research (AcSIR), CSIR-IHBT, Palmapur, India

^4^Present Address: Center of Innovative and Applied Bioprocessing (CIAB), Knowledge City, Sector-81, Mohali-140306, India.

*****Corresponding author: Yogendra S. Padwad ([yogendra@ihbt.res.in](mailto:yogendra@ihbt.res.in)); Tel.: 01894-233339 Extn. 473; Fax: +91-1894-230433

Sudesh K. Yadav ([sudesh@ciab.res.in](mailto:sudesh@ciab.res.in); skyt@rediffmail.com); Tel.: 0172-4990216; Fax: 0172-4990204

**Methods**

**Isolation of cellulose nanocrystals (CNCs).** CNCs were isolated from the leaves of *Syzygium cumini* by a combination of chemical and mechanical approach. Crushed leaves were given treatment with 3% (w/v) acidified NaClO_2_ (pH=3.5) for 3 h at 80°C with stirring. The material was then washed, re-soaked in acidified NaClO_2_ solution and kept overnight at room temperature. Then, alkali treatment with 3% KOH and 5% KOH was given for 2 h each at 80°C under constant stirring. Fiber at this stage was deemed as chemically pre-treated fiber (CPFs).To obtain pure CNCs, acid hydrolysis of CPFs was done by 65% (v/v) H_2_SO_4_ for 1 h at 45°C under vigorous stirring. The suspension was then diluted with excess of distilled water followed by centrifugation and dialysis until complete removal of acid. Thereafter, mechanical treatment using ultra-probe sonicator was given for 4, 8, 12, 20 and 30 min. The material after this stage was termed as cellulose nanocrystals (CNCs). The aqueous suspension of CNCs was characterized and freeze dried.

**Evaluation of microbicidal activity of nanocomposites (NCs)***.* The nutrient agar plates were seeded with 30 µL culture of these microbes (10^8^ CFU/mL). NCs hydrogels (50 mg wet weight) and strips (5 mm diameter) were taken as test samples, CNCs as negative control, and streptomycin (100 µg/mL) as positive control. The concentration of samples taken for antibacterial activity was decided on the basis of amount of Ag present. The plates were incubated overnight at 37 °C and then diameter of bacterial growth zone of inhibition (ZOI) was recorded. Anti-microbial activity of samples against each microbe was investigated for three times.

**Anti-bacterial mechanism of action of NCs.** For this, bacterial cells (10^8^ CFU/mL) of a gram positive (*S. aureus*) and a gram negative (*P. aeruginosa*) bacteria were incubated with wet weight of all the three NCs (50 mg/mL) for specific time intervals (0, 1, 2, 3, and 6 h) to observe the alterations in their shape, and then centrifuged. A drop of bacterial suspension from the pellet was placed on carbon coated copper grids and observed under TEM at appropriate magnification.

The protein leaky content in the supernatant obtained at 0 and 24 h of incubation of bacterial cells with NCs at 37 °C was determined by Bradford assay. The absorbance of bacterial cell supernatant was taken at 595 nm, and compared with the standard curve prepared from bovine serum albumin (0-10 μg/mL) to calculate protein leaky content.

***In vitro* cytotoxicity evaluation.** Keratinocytes isolated from mice were cultured in RPMI growth media supplemented with 10% FBS and 1% antibiotic-antimycotic and allowed to attain ~70% confluency. Keratinocytes were then trypsinized and approximately 20,000 cells in 100 μL of media were seeded into required number of wells in 96 well plates. The cells were allowed to adhere overnight by keeping in a 5% CO_2_ incubator at 37 °C. The materials to be tested were formulated at 25, 50 and 100 mg/mL (of wet weight of NCs) concentrations and the cells were treated with 100 μL of each in triplicates for 24 and 48 h. Post incubation, 50 μL of 50% trichloroacetic acid (TCA) was used to fix the cells at 4 °C for 1 h. This was followed by washing with water and air drying the plates. Further, 100 μL of SRB solution (0.4 % in glacial acetic acid) was added to each well and plates were placed in dark for 30 min. The unbound dye was then removed by proper washing with 1% glacial acetic acid. 10 mM tris base (100 μL/well) was used to dissolve the unbound dye and absorbance was taken at 540 nm in microplate reader (BioTeK Synergy H1 Hybrid Reader). To evaluate the percentage cytotoxicity of the test samples, absorbance values were used. The experiment was performed thrice to confirm the reproducibility of results.

**Diabetes induction in mice.** All the animals were fasted overnight before diabetes induction. An intra-peritoneal injection of streptozotocin (STZ; 80 mg/kg body weight dissolved in 0.1 M citric buffer at pH = 4.5) was given to mice. Immediately after STZ injection, water containing sucrose (10%) was given to mice to prevent sudden hypoglycemia shock. Venous blood glucose levels were monitored at regular intervals from samples drawn from ventral tail vein using a glucometer. After 72 h of first injection, a second dose of STZ (80 mg/kg body weight) was given to the mice following the same procedure as above. STZ injected mice were supervised regularly for any signs of undesirable symptoms/mortality, blood glucose levels and loss of body weight. Mice with glucose level (>200 mg/dL) were considered as diabetic. All animals included in this study developed hyperglycemia according to the mentioned inclusion criteria. The protocol optimized for diabetes induction using STZ at this dosage was found to be effective and led to stable induction of high glucose levels (>200 mg/dL) in mice with minimum/no mortality. Mice having stable high blood glucose levels were housed 1 week before commencing the wound healing experiment.

**Immunohistochemistry (IHC) procedure.** For immunohistochemical analysis, paraffin embedded wounded skin tissue sections (4 μm thick) of mice were mounted on poly-L-lysine coated slides, followed by deparaffinization and hydration. Antigen retrieval was done by exposing sections to sodium citrate buffer and quenching of endogenous peroxidases was performed using BLOXALL blocking solution (ImmPRESS excel staining kit, Vector Labs). The exposed sites were blocked by incubating sections with 2.5% normal horse serum. Sections were then incubated with a specific primary antibody at a particular dilution. Repeated PBS washes were given to remove unbound primary antibody followed by HRP-conjugated secondary antibody incubation. The sections were rinsed twice and incubated with DAB substrate^1^. Observations were done under bright field microscope in order to check the expression and activation status of platelet-derived growth factor (PDGF), basic fibroblast growth factor (b-FGF), vascular endothelial growth factor (VEGF), collagen I and collagen III for all the wounded skin tissue sections.


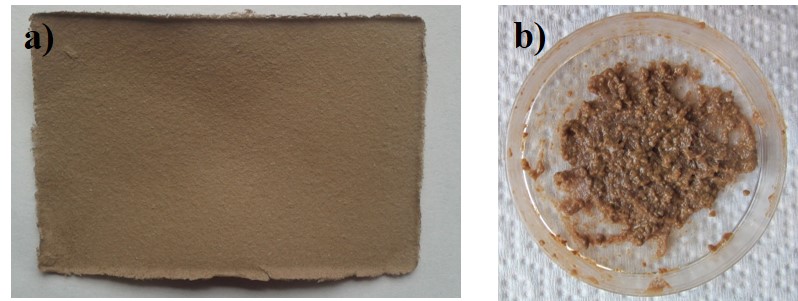


**Figure S1.** Photograph of (**a**) NCs strip and (**b**) NCs-ointment.

**Figure S2.** Scanning electron microscopic images representing the morphology of (**a**) untreated leaf, (**b**) fibers after bleaching treatment, and (**c**) fibers after alkali treatment.


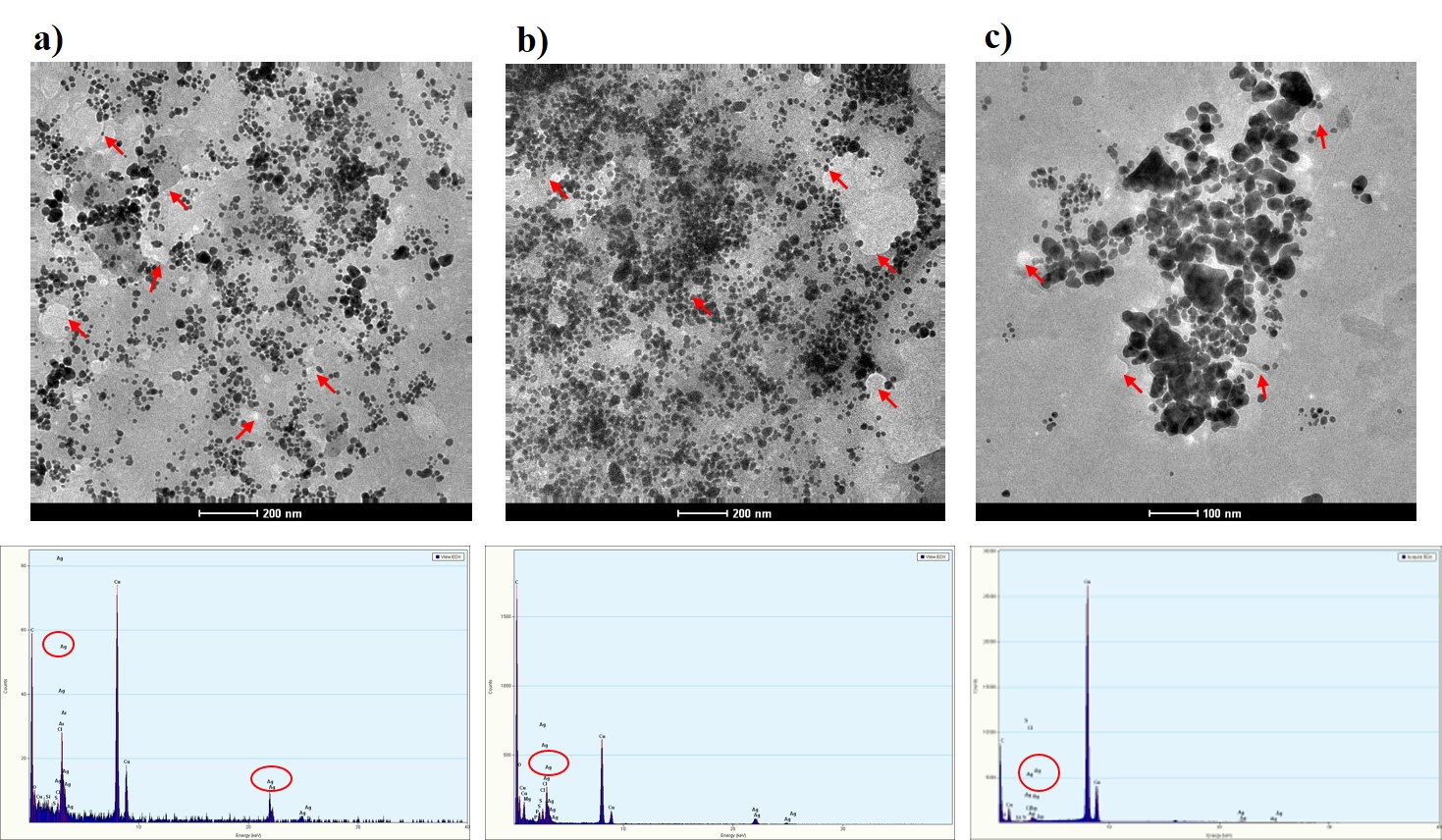


**Figure S3.** Enlarged TEM and TEM-EDX images of (**a**) NC-1, (**b**) NC-2 and (**c**) NC-3. Red colored arrows in TEM images show the presence of SC-CNCs matrix in the background and its porous nature, upon which AgNPs are impregnated or adsorbed. Red colored circles in TEM-EDX mark the peaks representing Ag element.


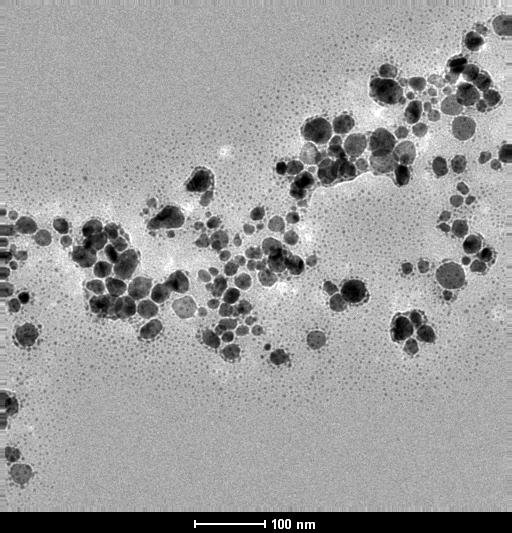


**Figure S4.** TEM image of bare AgNPs prepared from 1 mM silver nitrate solution using *S. cumini* leaf as reducing agent. AgNPs were of diameter 28±8 nm and used as control for wound healing studies.

**Figure S5.** Zeta potential measurements of SC-CNCs and NCs depicting the changes in surface charge values.

**Figure S6.** UV-Vis absorption spectra of NCs. The appearance of peak at ~ 420 nm confirmed the formation and presence of AgNPs in NCs.

**FTIR Spectroscopy Showing the Changes in Peak Intensity at Particular Wave number Indicate the Effect of Given Treatments during CNCs and NCs Formation**

FTIR spectra of untreated leaves and fibers obtained after bleaching treatment showed the existence of peak at 1510-1540 cm^-1^ corresponding to –C=C– stretch of aromatic rings of lignin, and a peak at 1730-1740 cm^-1^ attributed to acetyl and ester groups of hemicelluloses (Fig. S4). These peaks were absent in SC-CNCs, signifying the removal of lignin and hemicelluloses after chemical treatment^2^. The appearance of signal at 896 cm^-1^due to C-O stretching has confirmed the presence of cellulose in SC-CNCs^3^. A broad peak at 3500-3200 cm^-1^ was observed in SC-CNCs whereas peak intensity was decreased at this position in case of NCs. Decrease in intensity of this band in NCs suggested a strong interaction of primary hydroxyl groups of SC-CNCs with AgNPs through coordinate bonds during NCs formation. The intensity of significant band at 896 cm^-1^ further decreased in NCs as compared to SC-CNS which can be explained by presence of AgNPs on the cellulose surface.

**Figure S7.** Fourier transform infra-red spectra of (**a**) untreated leaf, (**b**) leaf fibers after bleaching treatment, (**c**) SC-CNCs, (**d**) NC-1, (**e**) NC-2 and (**f**) NC-3 showing the changes in surface functional moieties after chemical treatments.

**Figure S8.** X-ray diffraction spectra indicating the crystallinity index of (**a**) untreated leaf, (**b**) SC-CPFs, (**c**) SC-CNCs and (**d**) NCs.

**Figure S9.** Water uptake capacity (%) of SC-CNCs and NCs. Water holding capacity as a function of immersing time measures the efficiency of samples (as a wound dressing) to absorb wound exudate.


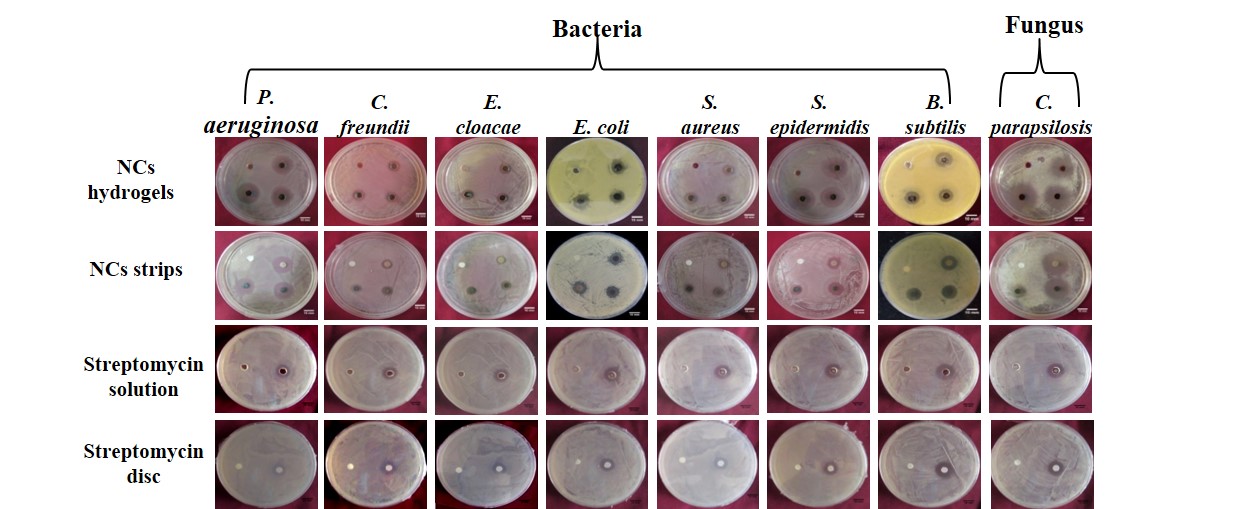


**Figure S10.** Picture showing the antimicrobial activity of NC-hydrogels and NC-strips against various gram negative as well as gram positive bacteria by well diffusion and disc diffusion method, respectively; where location of samples present in petriplates of NCs (hydrogels and strips) is CNCs control at top left, NC-1 at top right, NC-2 at bottom right, and NC-3 at bottom left.

**
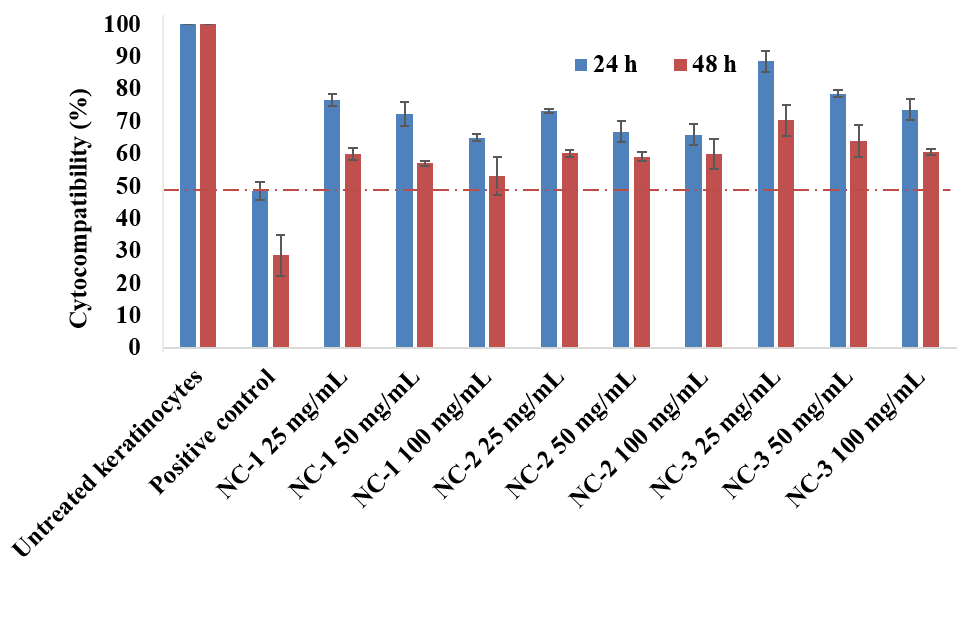
**

**Figure S11.** *In vitro* cytocompatibility studies of prepared NCs at 25, 50 and 100 mg/mL against primary mice keratinocytes after 24 and 48 h of treatment. The results are presented as mean± standard deviation (n = 3).

**
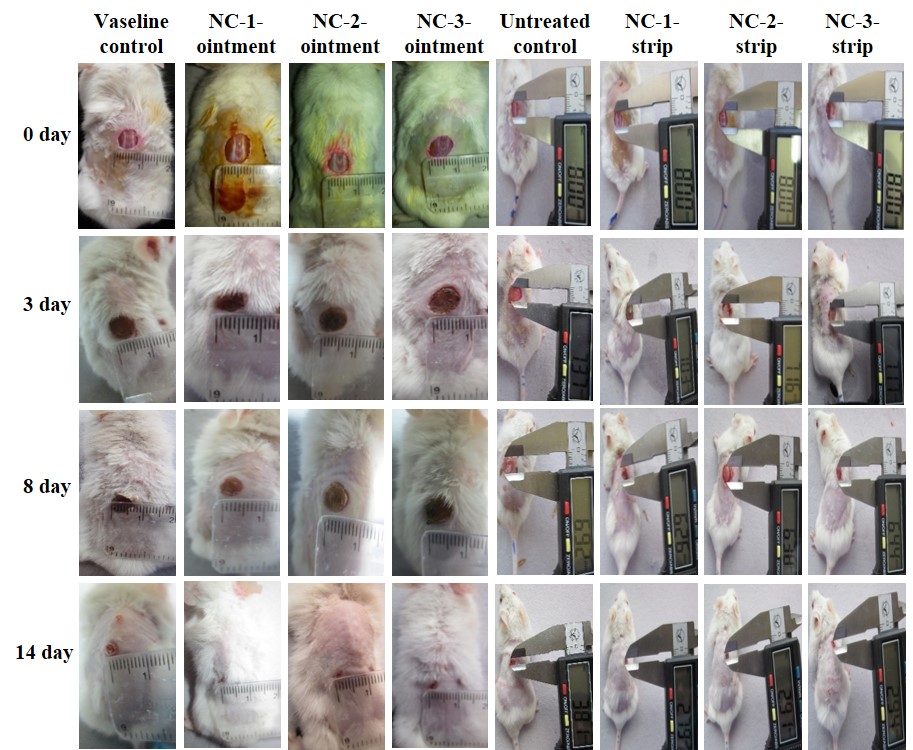
**

**Figure S12.** Digital camera photographs of wounds during therapy from day 0 to day 14 post wounding showing the effect of given treatment in *in vivo* acute wound healing mice model in a time dependent manner.


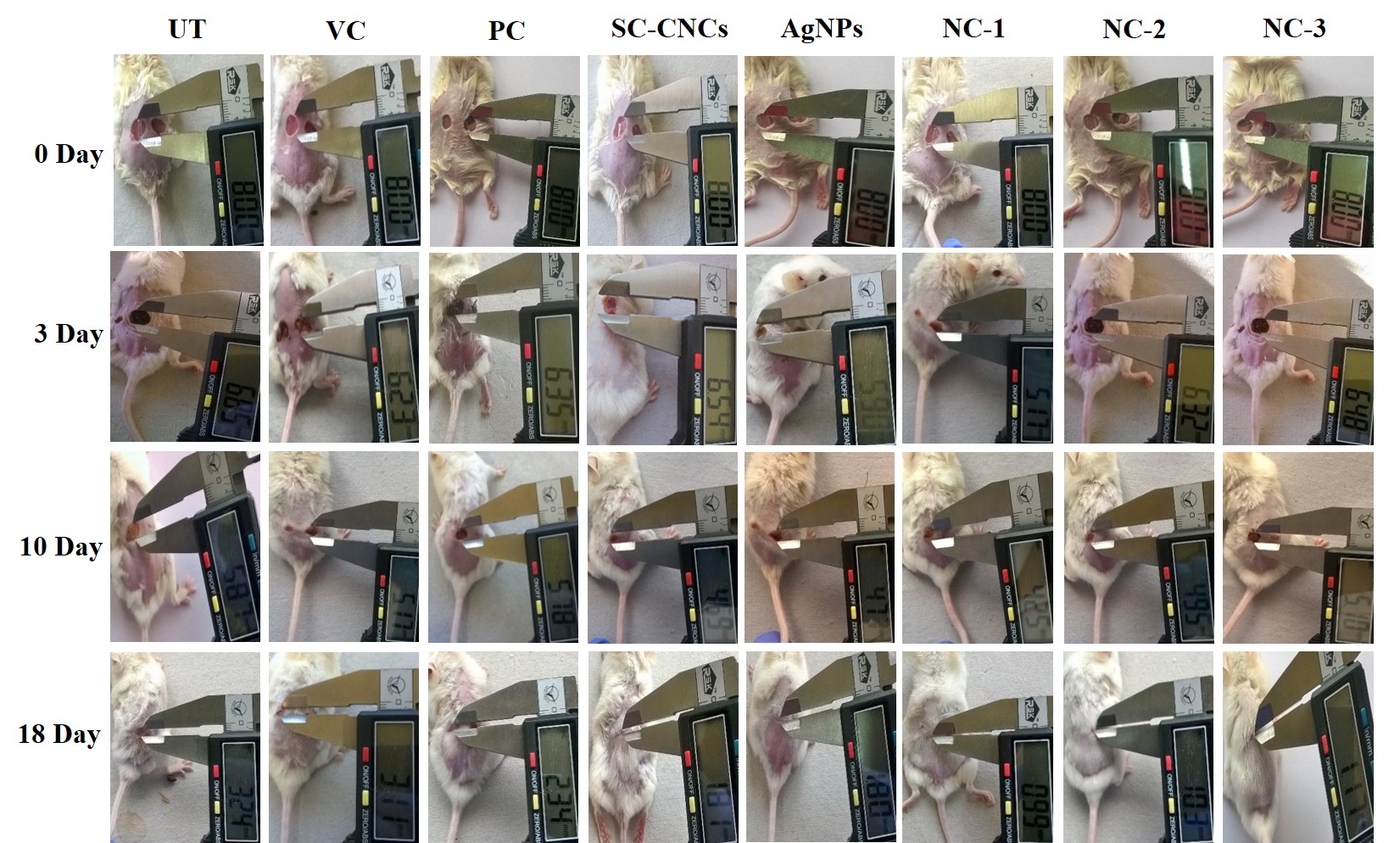


**Figure S13.** Digital camera photographs showing the measurement of wound diameter at specific treatment timelines from day 0 to day 18 post wounding to study the effect of given treatment in *in vivo* diabetic wound healing mice model in a time dependent manner.

**Figure S14.** Bright field micrographs of histopathological skin sections determining the changes in wound healing events of NCs treated and untreated mice by Masson’s trichome (M&T) stained images of wound tissues (**a**) at day 8 and 14 post wound in case of acute wound healing and (**b**) at day 10 and 18 post wound in diabetic wound healing experiments. The scale bars at the bottom of each image signify the size. Presence of blue color in M&T histopathology skin tissue sections indicate the collagen density.

**
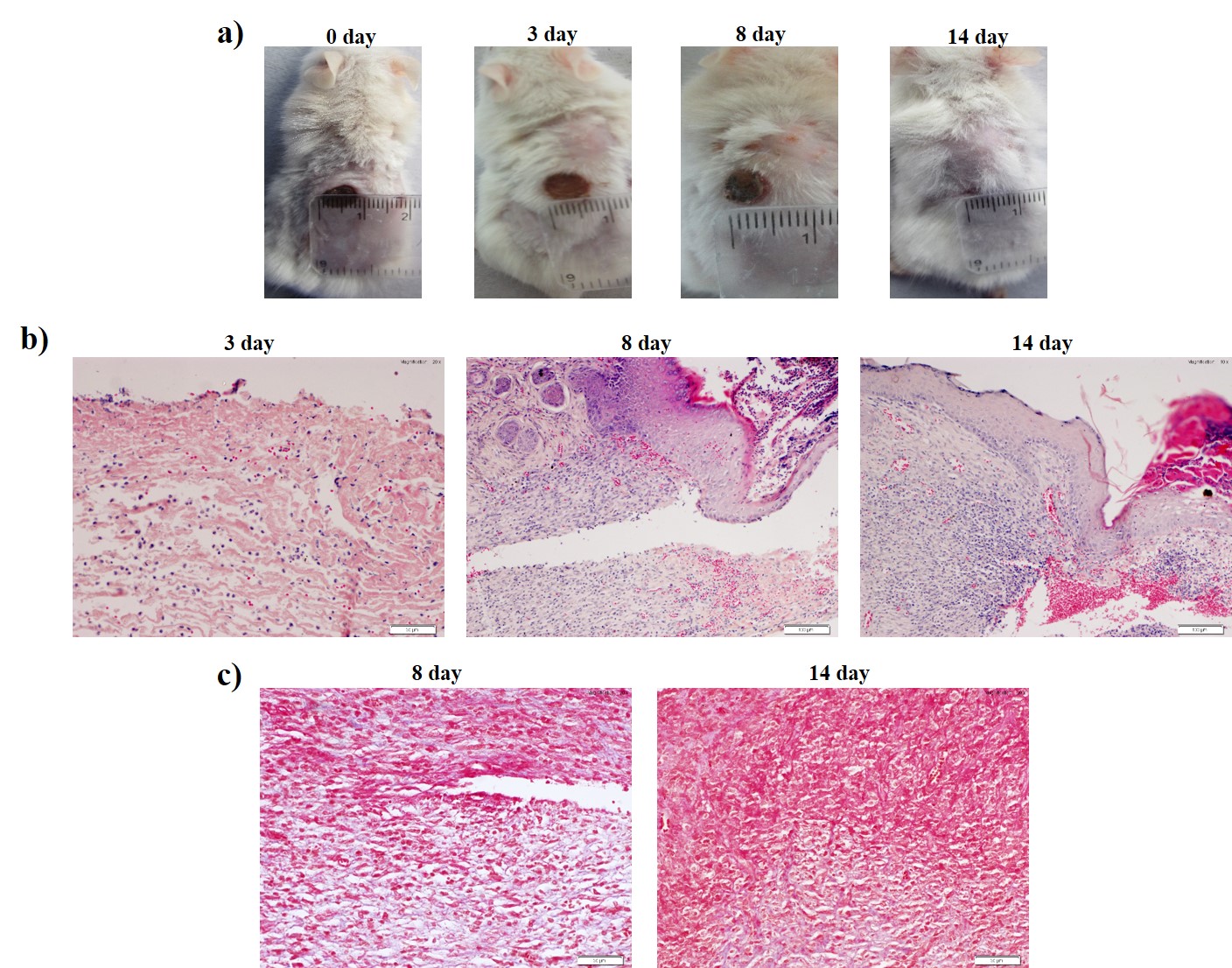
**

**Figure S15.** (**a**) Digital camera photographs of wound site of mice treated with AgNPs alone (control) at different time points. Bright field micrographs of stained skin tissue sections of AgNPs alone (control) treated mice using (**b**) haematoxylin and eosin (H&E) stain and (**c**) Masson’s trichome (M&T) stain in acute wound healing study.

**Figure S16.** Histograms represent level of protein expression (%) of (**a**) collagen 1, (**b**) collagen 3, (**c**) FGF, (**d**) PDGF and (**e**) VEGF in the wound tissue sections of diabetic mice on day 18 (collagen 1 and 3) and day 10 (FGF, PDGF and VEGF) post wound. The measurements are presented as mean ± standard deviation, n= 5 observation fields/group of respective IHC images. Groups not connected by same alphabets represent statistically significant differences at p < 0.05.

**Table S1.** Antibacterial activity of NCs (hydrogels and strips) showing bacterial zone of inhibition (ZOI in mm) after overnight incubation. The results are presented as mean ± standard deviation calculated from three independent measurements.

|  | **NC-1 hydrogel** | **NC-2**  **Hydrogel** | **NC-3**  **Hydrogel** | **NC-1**  **strip** | **NC-2**  **strip** | **NC-3**  **strip** | **Streptomycin**  **Solution** | | **Streptomycin**  **Disc** | |
| --- | --- | --- | --- | --- | --- | --- | --- | --- | --- | --- |
| ***P. aeruginosa*** | 21 ± 1 | 19 ± 1 | 20 ± 0.5 | 21 ± 1 | 19 ± 0.5 | 18 ± 1 | | 11 ± 0.5 | | 13 ± 0.2 |
| ***S. epidermidis*** | 23 ± 2 | 21 ± 1 | 22.5 ± 1 | 14 ± 1 | 12 ± 0.5 | 11 ± 1 | | 14 ± 0.5 | | 14 ± 0.3 |
| ***S. aureus*** | 15 ± 1 | 11 ± 0.5 | 11 ± 1 | 11 ± 2 | 9.5 ± 1 | 8 ± 1.5 | | 12 ± 1 | | 15 ± 1 |
| ***C. parapsilosis*** | 25 ± 1 | 22 ± 2 | 22 ± 1 | 26 ± 0.5 | 23 ± 1 | 15 ± 1 | | 12.5 ± 0.5 | | 14 ± 0.5 |
| ***C. freundii*** | 12 ± 1 | 11 ± 1 | 12 ± 0.5 | 11 ± 1 | 10 ± 0.5 | 9 ± 1 | | 14 ± 0.4 | | 13 ± 0.5 |
| ***E. cloacae*** | 11.5 ± 1 | 10 ± 0.5 | 10 ± 1 | 9.5 ± 1 | 9 ± 1 | 8 ± 0.5 | | 13 ± 0.5 | | 12 ± 0.2 |
| ***E. coli*** | 14.5 ± 1 | 12 ± 0.5 | 11.5 ± 1 | 14 ± 1 | 12 ± 0.5 | 12 ± 1 | | 14 ± 0.5 | | 15 ± 0.2 |
| ***B. subtilis*** | 14 ± 1 | 13 ± 0.5 | 11 ± 0.5 | 13.5 ± 0.5 | 11 ± 1 | 11 ± 0.5 | | 15 ± 0.3 | | 16 ± 0.1 |

**Table S2. Results showing the wound closure (%) and biochemical estimations of hydroxyproline, IL-6 and VEGF levels in mice treated with AgNPs alone (control) for acute wound healing at specific time intervals.**

|  | **% wound closure area** | **Hydroxyproline (ng/µL)** | **IL-6 (pg/mL)** | **VEGF (pg/mL)** |
| --- | --- | --- | --- | --- |
| **Day 3** | 36.3 ± 9 | 2.32 | 409.4 | - |
| **Day 8** | 62.5 ±8.5 | 2.03 | 184.75 | 69 |
| **Day 14** | 94.1±2.3 | 1.47 | 64.1 | 76.14 |

**References**

1. Gupta, M., Sharma, P., Mazumder, A. G., Patial, V. & Singh, D. Dwindling of cardio damaging effect of isoproterenol by *Punica granatum* L. peel extract involve activation of nitric oxide-mediated Nrf2/ARE signaling pathway and apoptosis inhibition. *Nitric Oxide* **50**, 105-113 (2015).
2. Chan, H.C., Chia, C. H., Zakaria, S., Ahmad, I. & Dufresne, A. Production and characterisation of cellulose and nano-crystalline cellulose from kenaf core wood. *BioResources* **8**, 785-794 (2013).
3. Xu, C., Zhu, S., Xing, C., Li, D., Zhu, N. & Zhou, H. Isolation and properties of cellulose nanofibrils from coconut palm petioles by different mechanical process. *PloS One* **10**, e0122123 (2015).
